# Efficacy and safety of finerenone in chronic kidney disease stages 3–4 in diabetic and non-diabetic patients with an eGFR 20–60 mL/min: a prospective cohort study

**DOI:** 10.3389/fneph.2026.1716452

**Published:** 2026-05-08

**Authors:** Hayder Aledan, Ammar Salih Abbood

**Affiliations:** 1 Nephrology, College of Medicine, Medicine, University of Basrah, Basrah, Iraq; 2 Cardiology, College of Medicine, Medicine, University of Basrah, Basrah, Iraq

**Keywords:** EGFR, finerenone, GLP1-ra, SGLT2I, UACR

## Abstract

**Background:**

Finerenone, when added to the standard-of-care treatment for chronic kidney disease (CKD), can effectively slow its progression. While previous trials have primarily focused on patients with diabetic kidney disease and an estimated glomerular filtration rate (eGFR) above 25 ml/min, this study aimed to evaluate the efficacy and safety of finerenone in patients with CKD stage 3–4, encompassing both diabetic and non-diabetic patients with an eGFR ranging from 20 to 60 ml/min.

**Methods:**

This prospective cohort study included 180 patients with CKD stage 3–4, including diabetic and non-diabetic participants. These patients had an eGFR of 20–60 ml/min and an urinary albumin-to-creatinine ratio (UACR) higher than 30 mg/g. The study was conducted between January 2023 and June 2024 at the Basra Nephrology and Transplantation Center and Private Clinic.

**Results:**

Finerenone showed statistically significant improvements in eGFR and UACR over a one-year period among all patient groups, including the subgroup of non-diabetic patients and patients with an eGFR between 20 and 25 ml/min (P < 0.001). Importantly, finerenone did not elevate serum potassium levels. The study population had a mean age of 61.67 ± 13.37 years, 50.6% aweremen,78% awerewdiabetic,22% awerewnon-diabeticand 65 (36%) hhadFR of 20–25 ml/min.

**Conclusions:**

Finerenone is a safe and effective approach for slowing the progression of CKD in patients with an eGFR > 20 ml/min and moderately to severely increased albuminuria, regardless of diabetes status.

## Introduction

Chronic kidney disease (CKD) progression is associated with increased morbidity and mortality (1). Pharmacological treatments, such as renin-angiotensin-aldosterone system inhibitors (RAASi), have been shown to slow the progression of CKD by decreasing intraglomerular pressure, thereby lowering glomerular hyperfiltration and blood pressure (2–4). Sodium-glucose cotransporter 2 inhibitors provide cardio-nephroprotection through multiple mechanisms, in addition to weight control, which has been shown to slow CKD progression (5). Glucagon-like peptide-1 agonists have also been shown to slow CKD progression and have recently been shown to exert nephroprotective effects against insulin and dipeptidyl peptidase-4-inhibitors (6). Kidney inflammation and fibrosis are among the pathways associated with CKD progression; therefore, finerenone, a selective non-steroidal mineralocorticoid receptor antagonist that targets these pathways by complementing existing therapies such as RAASi and SGLT2i, offers a multifaceted approach to slow the progression of CKD, particularly in diabetic kidney disease, but also in non-diabetic kidney disease (7). Renal benefits have been observed across various CKD stages, including stage 4 CKD with an eGFR >25 ml/min (8, 9). Hyperkalemia is a commonly observed adverse event; however, the rate of severe hyperkalemia and treatment discontinuation is very low (10).

The main objectives of this study were to assess the efficacy of finerenone on serum creatinine and UACR among patients with CKD stage 3–4 including later stage 4 (eGFR down to 20 ml/min) in diabetic and non-diabetic populations, and to assess the safety profile regarding the occurrence and severity of hyperkalemia.

## Methods

### Study design

This prospective cohort study included 180 patients with chronic kidney disease (CKD) stage 3–4 consulted the outpatient clinic at the Basra Nephrology and Transplantation Center and the Private Nephrology Clinic from January 2023 to June 2024. Patients aged ≥ 18 years with CKD stage 3–4 down to eGFR of 20 ml/min and urine albumin-creatinine ratio (UACR) >30 mg/g were included in the study; patients with eGFR <20 ml/min and UACR <30 mg/g were excluded. The study protocol, including the off-label use of finerenone in non-diabetic CKD patients, was reviewed and approved by the University of Basrah Ethics Committee. All participants provided written informed consent, which explicitly included information on the off-label use of finerenone.

### Data collection

Data were collected, including patient demographics, history of chronic diseases such as diabetes mellitus (DM), hypertension (HTN), coronary artery disease (CAD), heart failure with preserved ejection fraction (HFpEF), heart failure with reduced ejection fraction (HFrEF), CKD stages, and medications used, such as renin-angiotensin-aldosterone system inhibitors (RAASi), sodium-glucose cotransporter-2 inhibitors (SGLT2i), glucagon-like peptide-1 receptor agonists (GLP1-RA), loop diuretics, and thiazide diuretics. Baseline and 1-year eGFR, serum potassium, and UACR levels were documented.

### Definitions and measurements

The baseline estimated glomerular filtration rate (eGFR) was calculated using the CKD-EPI equation 2021 in the National Kidney Foundation application. Stage 3 CKD was defined as eGFR between 30–60 ml/min which was subdivided into stage 3a when eGFR was 45–60 ml/min and stage 3b when eGFR was 30–45 ml/min and stage 4 when eGFR was 15–30 ml/min but the lower limit eGFR for the present study was 20 ml/min (11). Serum creatinine levels were measured using the modified Jaffe colorimetric method. Serum K values were taken from the venous blood gas samples done with ABL800 machine. Hyperkalemia was defined as serum K levels > 5.5 mmol/l and hyperkalemia was graded as: mild 5.5-6.5 mmol/l, moderate 6.5-7.5 mmol/l and severe (> 7.5 mmol/l) (12). Urine ACR in spot urine samples was measured by immunonephelometry and graded as moderately increased albuminuria with UACR 30–300 mg/g and severely increased albuminuria with UACR >300 mg/g.

### Outcomes

The outcome of the study was to estimate the efficacy and safety of finerenone in patients with CKD stage 3–4 down to an eGFR of 20 ml/min in diabetic and non-diabetic patients on eGFR, UACR, and serum K levels over 1 year of therapy.

### Statistical analysis

The assumption of normality of continuous variables was analyzed using the Shapiro-Wilk test. Continuous normally distributed variables were analyzed using the mean ± standard deviation (SDs), and continuous skewed variables were analyzed using the median and interquartile range. Non-continuous variables were analyzed using numbers and percentages. Repeated measures ANOVA was used to estimate the effect of finerenone on eGFR, serum K, and UACR over 1-year of therapy in all cohorts and in subgroups of non-diabetic patients and those with eGFR of 20–25 ml/min. A line graph was used to assess the effects of SGLT2i and GLP1-RA on eGFR at 1-year. A scatter plot was used to assess the correlation between baseline UACR and eGFR at 1-year. Statistical significance was set at P < 0.05. Statistical analyses were performed using SPSS (version 28).

## Results

A total of 180 patients with CKD stage 3–4 were enrolled in this study. **Table 1** shows their baseline characteristics, with a mean age of 61.67 ± 13.37,years,mean BMI of 30.82 ± 5.76, 50.6% males and 49.4% females, 78% DM, 97.2% HTN, 52.8% CAD, 24.4% HFrEF, and 33.9% arding CKD staging, 58.9% hahadKD stage 3 (26.1% hahadtage 3a and 32.8% hahadtage 3b), and 41.1% hahadKD4. All patients were on different forms of RAASi, 73.3% on SGLT2i, 20.6% on GLP1-RA, 18.9% on both SGLT2i and GLP1-RA, 57.2% on loop diuretics, and 41.7% on thiazide diuretics. The baseline mean serum creatinine level was 2.2 ± 0.6, with a baseline mean eGFR of 31.5 ± 10 ml/min, and 36% hahadGFR between 20–25 ml/min. The baseline mean serum K was 4.76 0.67, and the baseline median UACR was 450 (45,1855), with 29.4% having UACR between 30–300 mg/g and 70.6% having UACR >300 mg/g.

**Table 1:** Baseline characteristics of the cohorts with CKD stage 3–4.

Baseline characteristics		Values
Age (years)		61.67 ± 13.37
Gender	Males	91 (50.6)
	Females	89 (49.4)
BMI		30.82 ± 5.76
DM		141 (78)
HTN		175 (97.2)
CAD		95 (52.8)
HFrEF		44 (24.4)
HFpEF		61 (33.9)
CKD stages	3	106 (58.9)
	3a	47 (26.1)
	3b	59 (32.8)
	4	74 (41.1)
RAASi use		180 (100)
SGLT2i use		132 (73.3)
GLP1-RA use		37 (20.6)
SGLT2i and GLP1-RA use		34 (18.9)
Loop diuretics use		103 (57.2)
Thiazide diuretics use		75 (41.7)
Potassium lowering agents use		0 (0.0)
Baseline serum creatinine mg/dl		2.2 ± 0.6
Baseline eGFR ml/min		31.5 ± 10
Baseline eGFR 20–25 ml/min		65 (36)
Baseline serum K levels mmol/l		4.76 ± 0.67
Baseline UACR mg/g		450 (45, 1855)
Baseline UACR mg/g	30-300	53 (29.4)
	>300	127 (70.6)

Values are expressed as mean ± SD, median (interquartile range) and n (%). BMI (body mass index); DM (diabetes mellitus); HTN (hypertension); CAD (coronary artery disease); HFrEF (heart failure with reduced ejection fraction); HFpEF (heart failure with preserved ejection fraction); CKD (chronic kidney disease); RAASi (renin-angiotensin-aldosterone system inhibitors); SGLT2i (sodium-glucose cotransporter-2 inhibitors; GLP1-RA (glucagon-like peptide-1 receptor agonists); K (potassium); UACR (urine albumin-creatinine ratio).

**Table 2** shows the statistically significant effect of finerenone at 1 year on eGFR and UACR in all cohorts (P <0.001) and subgroups of non-diabetic patients with eGFR of 20–25 ml/min (P <0.001) and non-statistically significant effect on serum K in all cohorts (P 0.906), in non-diabetic (P 0.776), and in eGFR 20–25 ml/min (0.817).

**Table 2:** Effect of finerenone on eGFR, UACR, and K levels after 1 year of therapy.

Characteristics	n (%)	Variable	Baseline	After 12 months	F	η^2^	P value
All cohorts	180 (100)	eGFR ml/min	32 ± 10	47 ± 15	413	0.698	<0.001
		UACR mg/g	542 ± 400	180 ± 170	360	0.668	<0.001
		K mmol/l	4.76 ± 0.7	4.77 ± 0.6	0.014	0.000	0.906
Cohorts with eGFR 20–25 ml/min	65 (36)	eGFR ml/min	21 ± 2	36 ± 8	187	0.746	<0.001
		UACR mg/g	602 ± 380	209 ± 152	159	0.713	<0.001
		K mmol/l	4.8 ± 0.7	4.8 ± 0.6	0.054	0.001	0.817
Cohorts with non-diabetes	39 (22)	eGFR ml/min	33 ± 10	50 ± 16	83	0.687	<0.001
		UACR mg/g	592 ± 431	203 ± 197	72	0.656	<0.001
		K mmol/l	4.7 ± 0.7	4.7 ± 0.7	0.082	0.002	0.776

Values are expressed as mean ± SD and n (%). A repeated-measures analysis of variance (ANOVA) was used for statistical analysis. Estimated glomerular filtration rate (eGFR), urine albumin-creatinine ratio (UACR), and potassium (K) levels.

**Figure 1:**
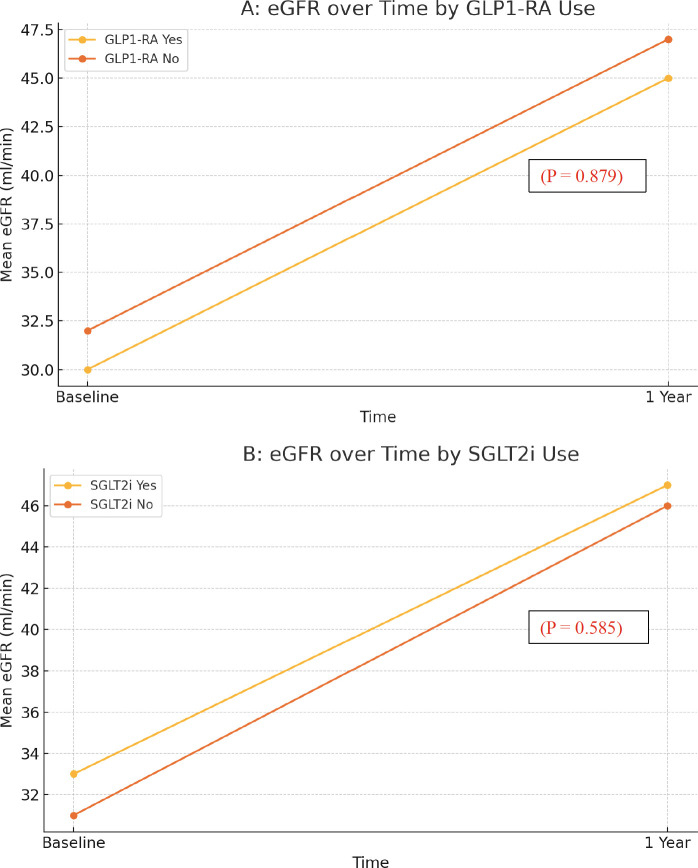
**(A)** Effect of GLP1-RA on eGFR at one year. **(B)** Effect of SGLT2i on eGFR at one year.

The effect of adjunctive therapies on eGFR was evaluated over a 1-year period using stratified line graphs. As shown in **Figure 1A**, patients receiving GLP1-RA exhibited a numerical improvement in eGFR compared with those not receiving GLP1-RA, although this difference was not statistically significant (P = 0.879). A similar trend was observed with SGLT2 inhibitors (**Figure 1B**), where patients on SGLT2i showed slightly higher eGFR values at 1 year compared to non-users, but again, without statistical significance (P = 0.585). These findings suggest that although these agents may have additive nephroprotective potential, their impact was not definitive in this cohort.

**Figure 2:**
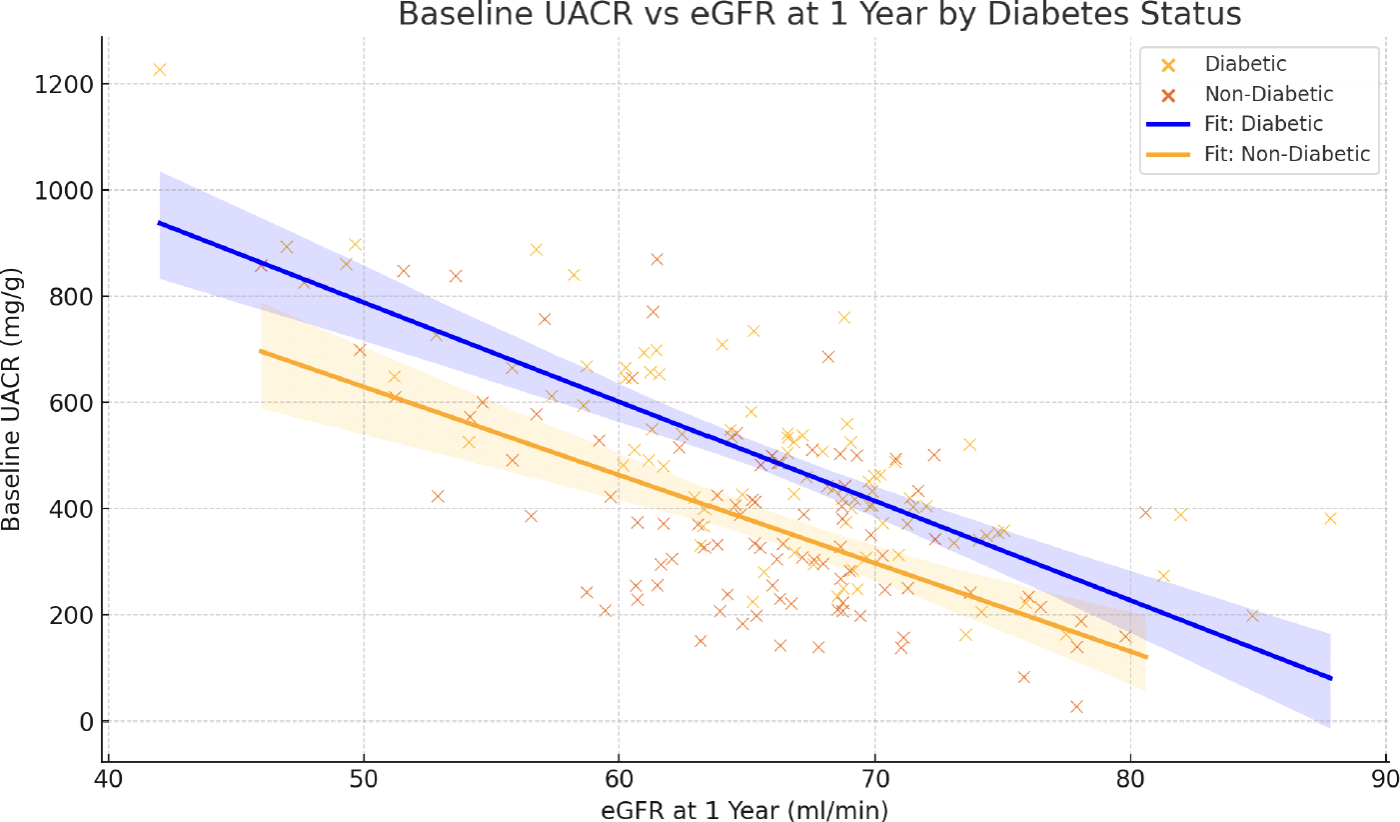
Scatter plot of baseline UACR by eGFR at 1 year.

To evaluate the relationship between baseline albuminuria and renal outcomes, a scatter plot was generated and stratified by diabetes status (**Figure 2**). A clear negative correlation was observed between the baseline UACR and eGFR at 1 year in both the diabetic and non-diabetic groups. The linear regression lines for each subgroup illustrate this inverse association, supporting the role of albuminuria as a predictor of disease progression. However, this correlation was more pronounced in patients with diabetes. These graphical analyses reinforce the prognostic relevance of albuminuria and provide further context for the observed therapeutic effects of finerenone.

## Discussion

This study aimed to assess the effects of finerenone on eGFR, UACR, and serum K levels in patients with CKD stage 3–4 including non-diabetic patients and those with an eGFR of 20–25 ml/min over 1 year period. The results indicated a significant efficacy and safety profile across the studied eGFR range of 20–60 ml/min and extended to non-diabetic patients. These findings suggest that the use of finerenone in patients with CKD stage 3–4 is safe and efficacious in slowing CKD progression. This aligns with previous studies by Bakris et al., Agarwal et al., and Goulooze et al., who found that finerenone in the background of standard-of-care treatment of CKD led to a sustained reduction in UACR and slowing of chronic eGFR decline (13–15). Similarly, a meta-analysis of randomized clinical trials by Chen et al. found both renal and cardiovascular benefits of finerenone with a low risk of hyperkalemia (16). A study by Sarafidis showed that the addition of SGLT2i or finerenone to standard-of-care treatment for albuminuric CKD reduced eGFR annular loss (17). The magnitude of eGFR improvement observed may reflect real-world variability, regression to the mean, or unmeasured confounding factors. Although the results suggest a potentially strong therapeutic effect, further controlled trials are required to confirm these findings. In the present study, the efficacy of finerenone was independent of the use of SGLT2i and GLP1-RA, suggesting that mineralocorticoid receptor overactivation, leading to kidney tissue inflammation and fibrosis, is an important pathway for CKD progression. This finding is aligned with a study by Rossing et al., who showed that the kidney and cardiovascular benefits of finerenone are independent of SGLT2i use (18, 19). The CONFIDENCE trial showed that combination therapy with finerenone and SGLT2i has additive effects, as they tackle different renal progression pathways (20). In this systematic review and meta-analysis, the use of finerenone or SGLT2i in the background of RAASi was associated with fewer kidney-specific composite outcomes (21). The renal benefits in patients with an eGFR of 20–25 ml/min without increasing the risk of hyperkalemia. A retrospective study by Mima et al. on the use of finerenone in patients with eGFR< 25 ml/min showed a statistically significant decrease in the slope of eGFR decline, with a non-significant reduction in proteinuria and no increase in serum K + levels over the study period (22). These results are aligned with those of a systematic review by Singh et al. and Ming-Zhu Zhang et al., who showed the cardiorenal benefits of finerenone but with a higher risk of hyperkalemia, which contradicts our study (23, 24). In the present study, the lower risk of hyperkalemia may also be due to the higher use of diuretics (nearly half of the patients), which was not studied in the systematic review by Singh et al. Hyperkalemia is a well-known adverse effect associated with mineralocorticoid receptor antagonists (MRAs) and a frequent cause of treatment discontinuation or underutilization in clinical practice. Notably, in our cohort, finerenone did not result in a statistically significant increase in serum potassium levels over one year, including in patients with advanced CKD (eGFR 20–25 ml/min) and those not on potassium-lowering therapies. This finding supports recent data suggesting that finerenone, as a non-steroidal MRA, may carry a lower hyperkalemia risk than traditional agents such as spironolactone or eplerenone. The absence of significant hyperkalemia across the subgroups is particularly reassuring, given the high baseline risk inherent in this population. These safety results may help alleviate provider hesitancy and support the broader adoption of finerenone in high-risk CKD populations. The results showed that finerenone is effective and safe in both diabetic and non-diabetic patients with CKD. This aligns with a previous study by Chen et al., which showed that finerenone is effective and safe in diabetic kidney disease (25). The FIND-CKD trial is a phase 3 trial targeting non-diabetic CKD patients, which strengthens our findings (26). We found that finerenone significantly reduced albuminuria, which is a marker of kidney damage due to diabetes, which is consistent with studies by Agarwal et al. and Zhou et al., who reported similar findings (27, 28).

The implications of this study are to implement the early use of finerenone in top-of-standard care treatment (RAASI, SGLT2i, and GLP1-RA) of CKD up to an eGFR of 20 ml/min, as it showed great nephroprotection and reduced eGFR decline over time without increasing the risk of hyperkalemia. The inclusion of both diabetic and non-diabetic patients allowed the benefits to be more generalizable across a broader CKD population.

The limitations of this study include the lack of a control arm, single-center study design, small sample size, and short follow-up period.

Future research should explore the use of finerenone in non-diabetic kidney diseases, including glomerular diseases and autosomal dominant polycystic kidney diseases, including patients with CKD and mildly increased albuminuria <30 mg/g.

In summary, this study highlights the efficacy and safety of finerenone in diabetic and non-diabetic CKD stage 3–4 down to an eGFR of 20 ml/min in terms of increased eGFR and albuminuria reduction without increasing the risk of hyperkalemia; therefore, we recommend implementing this strategy early with other pillars of nephroprotection.

## Data Availability

The raw data supporting the conclusions of this article will be made available by the authors, without undue reservation.
